# A neural network-based framework for enterprise financial error correction using AI and big data

**DOI:** 10.1038/s41598-026-48510-6

**Published:** 2026-04-16

**Authors:** Qasim Bndyan, Rediyar Salih, Khairi Ali Auso Ali, Kawar Mohammed Mousa, Dildar Haydar Ahmed

**Affiliations:** 1 Department of Business Administration, Near East University, Via Mersin 10, 99138 Nicosia, North Cyprus Turkey; 2 Department of Economic Science, University of Zakho, P.O. Box 12, Zakho, Iraq

**Keywords:** Enterprise management, Artificial intelligence, Big data, Financial anomaly detection, Cloud computing, Engineering, Mathematics and computing

## Abstract

The integration of artificial intelligence (AI) and big data technologies is transforming traditional business management practices. This study examines how AI-driven innovations improve decision-making, operational efficiency, and organizational adaptability in large enterprises. The paper examines the application of AI across various managerial domains, such as supply chain optimization, customer engagement, performance evaluation, and strategic planning, and identifies key opportunities for technological advancement, while also addressing critical implementation challenges. These include limitations in infrastructure, data integration, organizational culture, and workforce readiness. Through a conceptual framework supported by literature analysis and algorithmic modeling, the study describes how intelligent technologies can support real-time decision-making, automate complex processes, and support innovation. The paper also proposes a model for enterprise management that integrates cloud computing and neural network-based error correction for financial data, emphasizing the potential of AI to reduce operational risks and improve analytical accuracy. Overall, the research aims to contribute to a clearer understanding of AI’s strategic role in business management and offer a forward-looking perspective on its future development within enterprise systems. The study proposes a cloud-based enterprise management model integrated with a convolutional neural network (CNN)-based financial error correction mechanism, and the empirical evaluation shows strong anomaly-screening performance on the study dataset relative to the included classical baselines. In the empirical component of this study, “error correction” is operationalized as the detection and prioritization of abnormal financial-record patterns associated with default-risk outcomes, enabling audit and review workflows inside enterprise systems rather than automatic modification of ledger entries.

## Introduction

The convergence of big data and artificial intelligence (AI) has initiated a significant transformation in enterprise business management. In regions such as Asia, where digital infrastructure is rapidly expanding, these technologies are driving new waves of innovation, efficiency, and competitiveness^1–3^. Big data, with its defining characteristics of volume, variety, and velocity, enables organizations to respond more dynamically to complex market conditions^4,5^. This evolution has created a shift from static, reactive strategies toward data-driven, predictive, and agile business models^6,7^.

AI stands at the forefront of this shift. It enables firms to implement intelligent forecasting, automate routine decisions, and personalize customer experiences at scale^8–10^. Applications of AI now span across business functions, from human resources and finance to operations and marketing, improving precision and reducing operational lag^11–13^. However, the integration of AI technologies is not without obstacles. Many firms struggle with the challenges of data interoperability, employee readiness, security concerns, and the ethical effects of algorithmic decision-making^14–16^. In finance-focused applications, a major operational challenge is identifying abnormal or inconsistent patterns in accounting-related indicators early enough to prevent downstream reporting issues and risk exposures. Evidence from audit and accounting analytics shows that anomalies in enterprise records (e.g., unusual journal-entry patterns, unexpected account combinations, abnormal sign/amount relationships, or deviations from typical posting behavior) can be subtle, multi-factor, and embedded in high-dimensional general-ledger and transaction feature spaces, which makes rule-only validation checks insufficient in many realistic settings and motivates machine-learning–based detection pipelines that learn normal behavior and flag deviations^17^. Deep learning has been widely studied for anomaly detection, particularly where nonlinear relationships and complex feature interactions are prominent, and more recent finance-oriented studies increasingly emphasize pairing strong predictive models with explanation layers (e.g., SHAP-based local/global attributions) so that outputs can be communicated to stakeholders and reviewed in operational workflows^18,19^. At the same time, model transparency and auditability remain central concerns in regulated finance and accounting environments; therefore, contemporary auditing research highlights explainable-AI frameworks as a practical route for making AI-based judgments more defensible and reviewable during assurance and compliance activities^20^^,^^21^. Methodologically, recent progress in interpretable anomaly-detection modeling (including self-supervised learning approaches with built-in interpretability components) further supports the feasibility of capturing complex patterns while improving post-hoc understanding of model behavior^22^, although governance, documentation, and human oversight remain necessary in finance settings^23^. Within this context, the present work contributes a practical enterprise-oriented framework that positions neural detection as an Enterprise Management Information System (EMIS) integrated service supported by cloud infrastructure, while also reporting classification-based validation on a longitudinal enterprise dataset^24^.

**Fig. 1:**
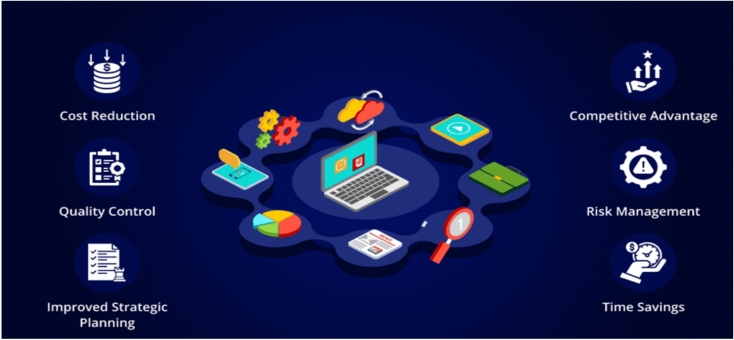
AI applications in strategic and operational layers of enterprise management.

Simultaneously, enterprises are transitioning toward digitally networked ecosystems. This shift fosters both horizontal integration—collaboration across industries—and vertical integration through intelligent supply chains, as illustrated in studies on enterprise alliances and cloud-based infrastructure^25–27^. Figure 1 reflects this dual function of AI in enabling both strategic foresight and operational control. AI enhances organizational learning, responsiveness, and customer alignment, marking a departure from conventional hierarchical models^28^.

**Fig. 2:**
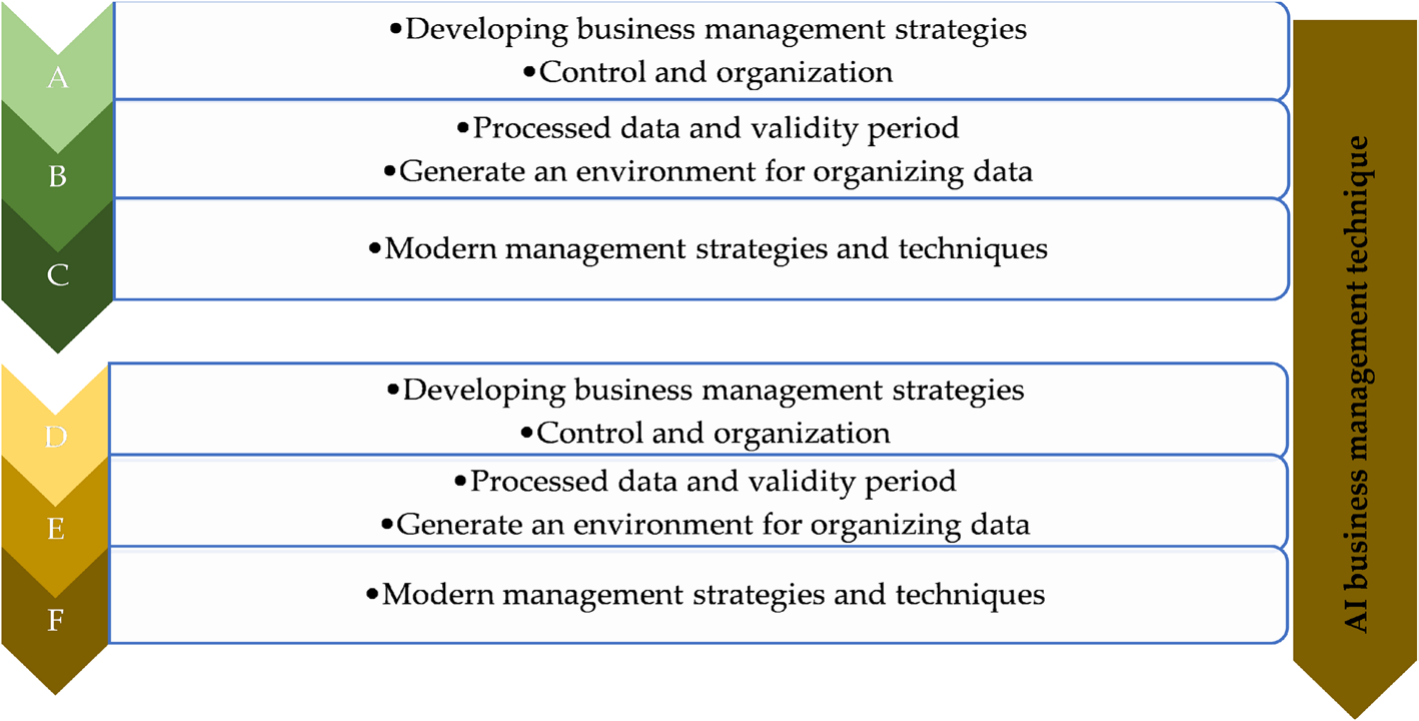
AI-enabled elements of the business management process.

Furthermore, Fig. 2 outlines the core dimensions of organizational management—planning, customer engagement, financial control, and resource coordination—all of which are now increasingly influenced by AI tools and data-driven platforms^29,30^. The integration of cloud computing, data warehouses, and enterprise information systems with AI adds further depth and scalability to these processes^31,32^.

Despite its promise, AI adoption often lacks a clear implementation roadmap. There remains a gap in aligning technical capabilities with strategic business goals, particularly in large enterprises dealing with legacy systems or fragmented data environments^33,34^. Addressing this gap, the present study examines how AI and big data can be effectively used for enterprise management. Specifically, it aims to: (1) review use cases of AI in core business functions; (2) evaluate technological integration pathways; and (3) propose a data-informed enterprise model grounded in neural networks and cloud platforms^35–37^.

## Materials and methods

As AI continues to evolve, its integration into enterprise management is no longer optional but essential. The aim of this study is to deepen the understanding of how AI and big data technologies can be effectively applied within large-scale business environments. Specifically, it seeks to identify the functional areas within enterprise management where AI technologies, such as machine learning, neural networks, and data-driven automation, can contribute to efficiency, cost reduction, and strategic agility^8,13,37^.

This research proposes a model that combines enterprise management information systems with AI-based algorithms for financial anomaly screening (detect–flag–review), operational forecasting, and customer behavior analysis. In this paper, ‘financial error correction’ refers to detecting abnormal indicator patterns linked to default-risk and routing them for review; it does not mean automatic modification of ledger values. The study also introduces a neural network-based method for identifying and flagging abnormal financial-record indicator patterns for review, which improves data integrity and decision accuracy^34,38^. In doing so, it provides applied findings on how organizations can improve their digital infrastructure and adapt management processes to the realities of the data economy^30,36^.

Additionally, the study uses empirical data from regional business databases over a 20-year period, including industrial, financial, and behavioral indicators. It builds and tests a classification model using a convolutional neural network (CNN) to detect and prioritize default-risk–linked abnormal indicator patterns for finance review, aiming to improve decision support and business intelligence capabilities^39^.

### Benefits of AI technologies in managing large enterprises

Artificial intelligence (AI) technologies have become integral to the modernization of large-scale business operations. Their ability to process vast volumes of data, identify patterns, and generate forecasts offers several significant advantages to enterprise-level management^8,36^. Below are the key areas where AI adds measurable value:

- Improved data analytics and decision-making
  AI enables real-time data processing and advanced analytics, allowing businesses to uncover trends and make more informed, data-driven decisions. Predictive algorithms help identify market opportunities and operational risks earlier than traditional systems^13,35^.
- Operational efficiency
  AI-powered automation reduces the need for manual intervention in routine tasks such as inventory control, scheduling, and workflow monitoring. This streamlining leads to lower operational costs and faster turnaround times^10^.
- Personalized customer experiences
  Machine learning models analyze customer behavior to deliver personalized content, product recommendations, and dynamic pricing strategies, raising satisfaction and retention^9,11^.
- Security and fraud detection
  AI systems can detect unusual patterns in transactions or system behaviors, helping prevent cyber threats and financial fraud through real-time alerts and automated risk assessments^30,34^.
- Human resource optimization
  AI supports HR by streamlining recruitment, automating employee engagement analysis, and predicting workforce needs based on performance metrics and company trends^12^.
- Cost reduction and resource allocation
  Through optimization of supply chains, predictive maintenance, and dynamic resource planning, AI reduces waste and improves the allocation of financial and human capital^14,37^.
- Innovation in product development
  AI supports creativity by identifying unmet customer needs and generating prototypes based on consumer feedback and market analysis, thus accelerating innovation cycles^39^.

In summary, the adoption of AI technologies enables large enterprises to transition from reactive to proactive strategies, which improves agility, productivity, and resilience in a competitive digital economy.

### The proposed method

Effective integration of AI into enterprise business management requires a data-driven framework that combines modern computer networks, enterprise management information systems, and cloud computing technologies. The method is designed to collect, store, and analyze large volumes of enterprise data, with the goal of improving operational efficiency, decision-making accuracy, and responsiveness to market changes^36,37^.

**Fig. 3:**
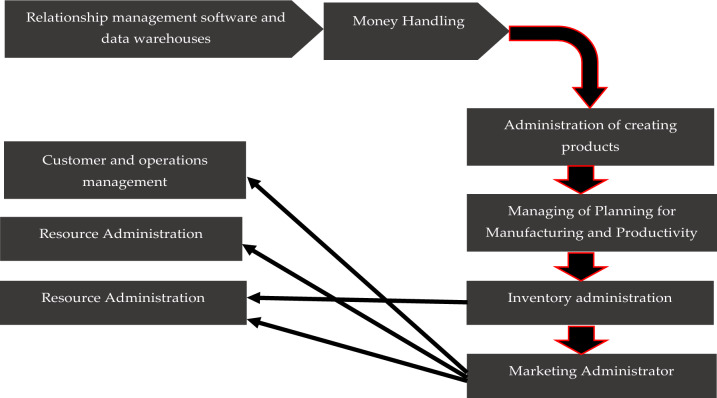
AI-supported construction model of enterprise information systems built on cloud architecture.

At the core of this framework is the EMIS, which consolidates data from various business processes, including product development, procurement, production, sales, finance, and customer service. Process mapping and analysis support the identification of inefficiencies and the automation of repetitive tasks, resulting in more agile operations^7,12^. As shown in Fig. 3, this model utilizes cloud computing to virtualize infrastructure and applications, allowing for real-time access to critical business data^40^.

The architecture includes three core layers:

- Infrastructure layer: Provides virtualized servers and storage, enabling scalable resource use^30^.
- Platform layer: Supports software development, testing, and deployment using APIs, databases, and computing tools^32^.
- Application layer: Hosts business-critical applications such as inventory control, finance, customer relationship management (CRM), and performance analytics^29^.

Platform alignment with business needs is supported through adaptive load balancing, security protocols, and business intelligence tools for KPI tracking and forecasting^10^.

The proposed CNN-based correction mechanism is deployed as an application-layer “Financial Data Quality Service” that interacts with EMIS through standardized APIs. In practical terms, operational systems (ERP/CRM/procurement/sales) write transactions and master data to the EMIS database or data warehouse. A scheduled or near-real-time ingestion job then extracts the required financial and industrial indicators (83 variables in this study) into a feature payload that is sent to the correction service. The correction service returns a risk/anomaly decision and an auditable scoring record (timestamp, model version, input fingerprint/hash, output score, and class label) that is stored in the EMIS audit repository and surfaced to finance users for review.

A typical data flow follows four stages:

1. Ingestion: EMIS exports a standardized record (entity ID, period, indicator vector) to the model endpoint.
2. Scoring: the correction service normalizes the record using the same transformations fitted on training data, runs CNN inference, and produces a decision score and class label.
3. Audit logging: the service writes an immutable log entry to the EMIS audit store (timestamp, model version, input hash, output score, and reviewer status).
4. Correction workflow: finance users receive the flagged record inside EMIS (dashboard/task queue). If a correction is approved, EMIS updates the record status (e.g., “verified default-risk anomaly” vs. “cleared”), and the decision is preserved for compliance and traceability.

The system can be implemented through RESTful APIs at the platform layer^29,32^. A minimal interface includes:

- POST /ingest Financial Record: accepts {entity_id, period, indicators [83], source_system, record_version}.
- POST /score Anomaly: returns {probability, class_label, confidence, model_version}.
- POST /audit Log: stores {entity_id, period, input_fingerprint, output, reviewer_action}.

This explicit integration closes the gap between the cloud/EMIS framework and the CNN-based correction algorithm, showing how financial records move through enterprise systems and how the model outputs are operationalized in accounting review and governance.

**Fig. 4:**
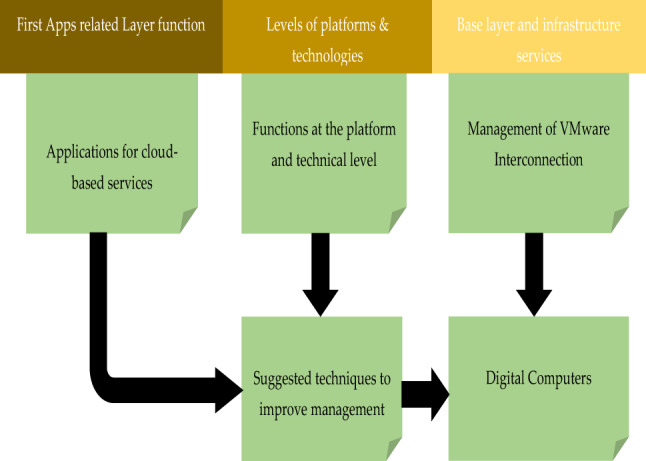
Integration of AI and big data in enterprise leadership and decision-making processes.

Figure 4 demonstrates the flow of business leadership functions underpinned by AI and big data, reinforcing how intelligent systems contribute to value creation, risk mitigation, and strategic alignment^41^.

The framework also includes a neural network-based algorithm for identifying abnormal enterprise indicator patterns and routing high-risk cases into an auditable review workflow inside EMIS. In this study, the CNN screens structured financial/industrial indicators and flags default-risk–linked irregular patterns for prioritization and human review, rather than automatically modifying accounting ledger entries^34,39^. The system is trained on a multi-year dataset containing financial, industrial, and behavioral indicators, ensuring robustness across varied business scenarios^42^.

The proposed method modernizes internal data processing and enables dynamic customer management, production forecasting, and resource optimization. It bridges the gap between AI theory and business application, positioning AI as a usable tool for digital transformation in enterprise management^8,14^.

### Neural network-based correction algorithm

In enterprise financial-control environments, “error correction” is most safely and realistically implemented as a detect-flag-review process rather than an automatic rewriting of accounting ledgers. Accordingly, this study operationalizes financial error correction as the detection and prioritization of abnormal financial-record indicator patterns that are associated with default-risk outcomes, and the routing of those high-risk records into an auditable finance-review workflow within the EMIS. The model therefore supports correction decisions (audit/review, verification, and controlled amendment in EMIS) instead of directly generating corrected numeric values^43^.

#### Problem formulation and notation

Each observation corresponds to one enterprise entity in one reporting period (e.g., customer-period) represented by a fixed-length vector of structured indicators extracted from EMIS and associated data sources (financial variables, industrial indicators, and macroeconomic/context variables). Let the dataset be.

1 $$\mathcal{D}=\{\left({x}_{i},{y}_{i}\right){\}}_{i=1}^{N}, {x}_{i}\in {\mathbb{R}}^{d}, {y}_{i}\in \{\mathrm{0,1}\}$$

In this study, d = 83 indicators are used per record and N = 6919 total records are available. The binary label is defined consistently with the Results section:

- ${y}_{i}=1$: defaulted (interpreted here as a high-risk / abnormal financial-indicator pattern requiring prioritization for review),
- ${y}_{i}=0$: non-defaulted (interpreted here as a baseline / normal pattern under the observed data distribution).

The model learns a scoring function $f\left(\cdot \right)$ that outputs the probability of default risk:

2 $${\widehat{p}}_{i}=f\left({x}_{i}\right)\approx {\mathbb{P}}\left({y}_{i}=1\mid {x}_{i}\right)$$

During deployment, records with high p ^ are flagged and sent to the EMIS review queue; the system does not automatically change ledger entries^44^.

#### Covariance-stabilized normalization for high-dimensional financial indicators

Enterprise financial indicators are typically heterogeneous (different scales, units, and correlations). To ensure stable learning and to match the “standardized inputs” assumption used consistently across the CNN and baseline models, a training-fitted normalization is applied.

Stack the feature vectors into a matrix:

3 $$X = \left[ {\begin{array}{*{20}c} {x_{1}^{{\mathrm{T}}} } \\ {x_{2}^{{\mathrm{T}}} } \\ \vdots \\ {x_{{\mathrm{N}}}^{{\mathrm{T}}} } \\ \end{array} } \right] \in \mathbb{R}^{{N \times d}}$$

Compute the empirical mean vector and covariance matrix on the training subset only (to avoid leakage):

4 $$\mu = \frac{1}{{N_{{{\mathrm{train}}}} }}\mathop \sum \limits_{{i \in {\mathrm{train}}}} x_{i} ,\,\,\,\,\Sigma = \frac{1}{{N_{{{\mathrm{train}}}} }}\mathop \sum \limits_{{i \in {\mathrm{train}}}} \left( {x_{i} - \mu } \right)\left( {x_{i} - \mu } \right)^{{\rm T}}$$

A Cholesky factorization provides a numerically efficient “square-root” factor:

5 $${\Sigma } = {{HH}}^{\rm T}$$

where H is lower triangular.

Each record is then transformed to a stabilized/whitened representation:

6 $${\widetilde{x}}_{i}={H}^{-1}\left({x}_{i}-\mu \right)$$

This transformation (i) reduces instability from different indicator scales, and (ii) mitigates collinearity among financial ratios and macro/industrial indicators. Importantly, ${\widetilde{x}}_{i}$ is a modeling representation, not a replacement of the enterprise record. The original values remain unchanged in EMIS; the transformed representation is used only for scoring and pattern detection.

#### Optional indicator association screening for interpretability

To support indicator-level diagnostic insight (e.g., which indicators are statistically associated with the default-risk label), a chi-square association test can be applied as an analysis layer. Because chi-square requires categorical values, each continuous indicator is discretized into $B$ bins (e.g., quantiles). For a given indicator, let ${O}_{ab}$ be the observed count in bin $a$ and class $b$, and let ${E}_{ab}$ be the expected count under independence. The chi-square statistic is.

7 $${\chi }^{2}=\sum_{a=1}^{B}\sum_{b=1}^{2}\frac{{\left({O}_{ab}-{E}_{ab}\right)}^{2}}{{E}_{ab}}$$

Expected counts are computed from marginal totals:

8 $${E}_{ab}=\frac{{R}_{a}{C}_{b}}{n}$$

where ${R}_{a}=\sum_{b}{O}_{ab}$, ${C}_{b}=\sum_{a}{O}_{ab}$, and $n=\sum_{a,b}{O}_{ab}$. Larger ${\chi }^{2}$ values indicate stronger dependence between the indicator and the label.

To avoid contradictions with the reported experiments, the chi-square analysis is used for ranking and interpretation, not as a mandatory feature-elimination step; the CNN and baselines can still be trained using the full 83-indicator vector.

#### 1D CNN for tabular enterprise indicators

Although convolutional neural networks are often introduced for images, here the CNN is used as a 1D feature-interaction extractor over a finance-informed ordered indicator vector. Each normalized record $\widetilde{x}\in {\mathbb{R}}^{d}$ is treated as a 1D sequence of length d. The indicator order is fixed across training, testing, and deployment as follows: macroeconomic and industrial-context indicators first, followed by firm-level financial indicators arranged in related accounting blocks, and finally the control/behavioral indicators. Within the financial block, conceptually related indicators are kept adjacent so that 1D convolution kernels operate over locally meaningful neighborhoods rather than an arbitrary permutation of columns. This design gives an economic rationale for local receptive fields and supports interpretation of learned interactions at the indicator-block level.

Let $\tilde{x} = \left[ {\tilde{x}_{1} , \ldots ,\tilde{x}_{d} } \right]^{\rm T}$ . For convolution layer ${\ell}$ with filter $i$ of kernel size ${k}_{{\ell}}$, weights ${w}_{{\ell},i}\in {\mathbb{R}}^{{k}_{{\ell}}}$, and bias ${b}_{{\ell},i}$, the pre-activation at position $j$ is

9 $${s}_{{\ell},i,j}=\sum_{u=0}^{{k}_{{\ell}}-1}{w}_{{\ell},i,u}\hspace{0.17em}{\widetilde{x}}_{j+u}+{b}_{{\ell},i},\,\, j=1,\dots ,d-{k}_{{\ell}}+1$$

A ReLU nonlinearity produces the feature-map activation:

10 $${h}_{{\ell},i,j}=max\left(0,{s}_{{\ell},i,j}\right)$$

Across ${n}_{f}$ filters, the resulting feature maps are pooled to reduce sensitivity to local noise and to concentrate the strongest evidence of abnormal patterns. A segment-based max pooling strategy is used: each feature map is divided into three contiguous segments and the maximum is taken from each segment.

11 $${\widehat{h}}_{{\ell},i}=\left[\begin{array}{c}max\left({h}_{{\ell},i}^{\left(1\right)}\right)\\ max\left({h}_{{\ell},i}^{\left(2\right)}\right)\\ max\left({h}_{{\ell},i}^{\left(3\right)}\right)\end{array}\right]$$

Pooled outputs from all filters (and potentially multiple convolutional blocks) are concatenated into a representation vector $z$, which is passed to the final classifier head.

#### Output layer, loss function, and training protocol

Because the task is binary classification (defaulted vs. non-defaulted), the model produces two logits $o=\left[{o}_{0},{o}_{1}\right]$ and a SoftMax probability vector:

12 $$\widehat{\pi }={\mathrm{SoftMax}}\left(o\right),\,\, {\widehat{\pi }}_{c}=\frac{{e}^{{o}_{c}}}{{e}^{{o}_{0}}+{e}^{{o}_{1}}}, c\in \left\{\mathrm{0,1}\right\}$$

The predicted default-risk score is $\widehat{p}={\widehat{\pi }}_{1}$. Training minimizes categorical cross-entropy over mini-batches (consistent with the Results section’s description).

13 $${\mathcal{L}} = - \frac{1}{B}\sum\limits_{{a = 1}}^{B} {\sum\limits_{{c \in \left\{ {{\mathrm{0,1}}} \right\}}} 1 } \left[ {y^{{\left( a \right)}} = c} \right]\log \left( {\hat{\pi }_{c}^{{\left( a \right)}} } \right) + \lambda \left\| \theta \right\|_{2}^{2}$$

where $B$ is the batch size, $\theta$ denotes all trainable parameters, and $\lambda \ge 0$ controls ${L}_{2}$ regularization.

Because enterprise default datasets often exhibit class imbalance, the experimental protocol matches the paper’s Results section: the train–test split is performed first, then SMOTE is applied only to the training subset to obtain a 1:1 class ratio, while the held-out test set remains unchanged. This ensures the evaluation reflects real held-out performance and avoids data leakage^45^. For uncertainty reporting, the study computes 95% confidence intervals on the held-out test metrics; these intervals are intended to capture sampling uncertainty on the fixed test set and do not replace multi-seed repetition for quantifying training stochasticity.

#### Detect–flag–review in EMIS

To operationalize correction as a controlled enterprise process, the CNN is deployed as an application-layer Financial Data Quality Service (as described in the Proposed Approach section)^46^. For each incoming record:

1. Ingestion: EMIS exports $\{entity\_id,period,x\}$ (83 indicators in a fixed order).
2. Normalization: The service applies the training-fitted $\left(\mu ,H\right)$ transform to compute $\widetilde{x}$ (Eq. 6).
3. Scoring: The CNN produces $\widehat{p}={\mathbb{P}}\left(y=1\mid x\right)$ (Eq. 12).
4. Flagging rule: A threshold $\tau$ (or a top-$K$ policy) converts $\widehat{p}$ into an operational label:
  if $\widehat{p}\ge \tau$: record is flagged as high-risk anomaly candidate,
  else: record is marked as low-risk for this screening stage.
5. Audit logging: The service writes an immutable log entry (timestamp, model version, input fingerprint/hash, $\widehat{p}$, threshold decision, reviewer status).
6. Human review and controlled update: Finance users review flagged records inside EMIS and decide whether the record should be corrected/adjusted in the accounting system or cleared as valid. The system preserves both the model output and the reviewer’s action for traceability.

## Results and discussions

To evaluate the performance of the proposed CNN model, a comprehensive experiment was conducted using a 20-year regional enterprise dataset comprising 6,919 samples. The dataset included 83 total indicators, 31 industrial or macroeconomic indicators, 50 financial variables, and 2 additional control/behavioral indicators, and 1 binary classification label indicating whether an enterprise (firm) record was defaulted or non-defaulted. In this study, the term “financial error” refers to abnormal or inconsistent indicator patterns that are associated with default-risk outcomes and therefore require prioritization for finance review and auditing within enterprise information systems, rather than automatic rewriting of accounting ledgers. As shown in Table 1, the dataset was divided into a training set and a test set, with class balancing applied to correct the inherent sample distribution disparity.

**Table 1:** Sample allocation of financial records.

Category	Training set	Test set	Total
Non-defaulted samples	3000	640	3640
Defaulted samples	3000	279	3279
Total samples	6000	919	6919

The dataset exhibits class imbalance in its original form, which is common in financial datasets. To address this during model training, the train–test split was performed first, and class balancing was applied only to the training subset using the Synthetic Minority Over-sampling Technique (SMOTE) in MATLAB R2015a. Table 1 reports the post-balancing training composition (3000 non-defaulted and 3000 defaulted), while the held-out test set (919 records) was kept unchanged and was not oversampled^12,38^. For fairness across models, all baselines (SVM, Naive Bayes, and LDA) were trained using the same training-fitted normalization used for CNN inputs (mean-centering and covariance-stabilized whitening; Eq. 6). SVM hyperparameters were tuned via cross-validated search over C and kernel parameters; LDA used covariance regularization/shrinkage options; Naive Bayes was configured with distribution assumptions consistent with standardized continuous indicators.

The CNN model was implemented with multiple convolutional layers, each followed by ReLU activation and max-pooling, to extract locally interacting patterns from ordered financial indicator vectors. Unlike traditional models that often rely on manual feature engineering, the CNN model learns internal feature compositions from the accounting and industrial indicators, which supports generalization to unseen samples^34^. The model was optimized using backpropagation with categorical cross-entropy as the loss function and a SoftMax output layer for binary classification^47^.

Baseline models were selected to represent widely used classical classifiers for tabular finance data (SVM, Naive Bayes, and LDA) and to provide an interpretable point of comparison for the proposed CNN. At the same time, modern gradient-boosted decision tree ensembles such as XGBoost^48^, LightGBM^49^, and CatBoost^50^, are widely recognized as strong baselines for structured/tabular classification, and recent benchmarking studies continue to show that tree-based methods are often highly competitive or superior on medium-sized tabular datasets^51^. Because these additional baselines were not executed on the present dataset, the empirical results reported here should be interpreted as demonstrating performance relative to the included classical baselines only, rather than as establishing superiority over the current strongest tabular ensemble methods. Accordingly, extended benchmarking against XGBoost, LightGBM, and CatBoost is identified as a necessary next validation step for this framework.

**Fig. 5:**
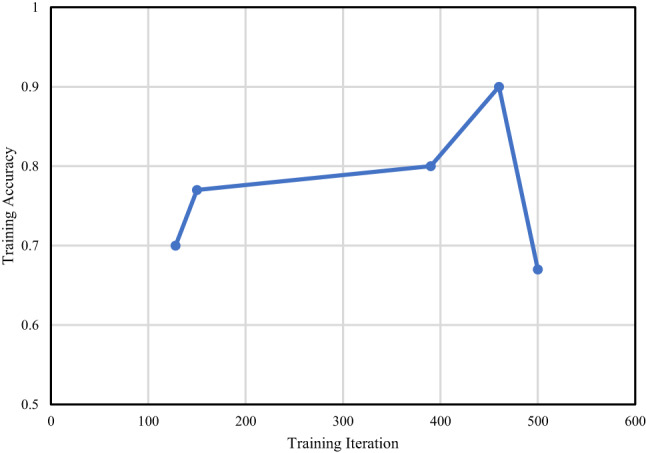
CNN training accuracy across training iterations.

Figure 5 illustrates the training performance of the CNN across iterations. The model’s classification accuracy improved significantly with the introduction of deeper convolutional layers, showing the contribution of hierarchical feature abstraction. The final training accuracy exceeded 95%, and the held-out test-set performance is reported below to reflect generalization under the described evaluation protocol.

**Fig. 6:**
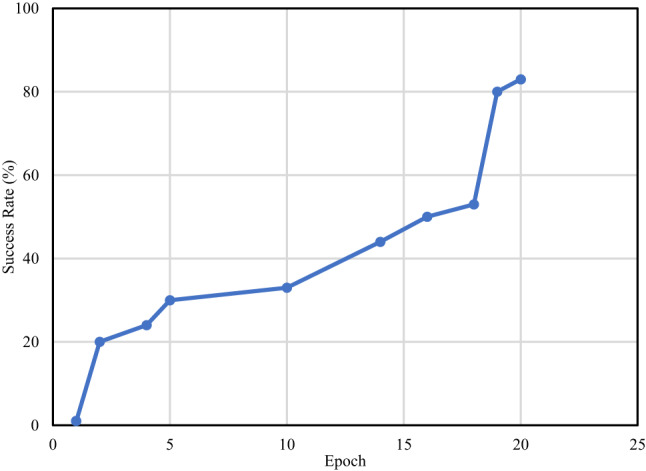
CNN performance trend across training epochs.

When benchmarked against standard machine learning models, including Support Vector Machine (SVM), Naive Bayes (NB), and Linear Discriminant Analysis (LDA), the CNN outperformed all baselines in key performance metrics. As shown in the performance analysis presented in Fig. 6, the model achieved the following:

- G-mean: 0.93
- Type I error (false positive rate): 4.2%
- Type II error (false negative rate): 5.6%
- Balanced accuracy (B-M): 92.5%
- Matthews correlation coefficient (MCC): 0.89
- Area under the curve (AUC): 0.96

Using the held-out test composition (non-defaulted n0 = 640, defaulted n1 = 279) and the reported AUC of 0.96, the 95% confidence interval for AUC is [0.944, 0.976], computed with the Hanley–McNeil approximation for ROC-area sampling variance^52^. Using the held-out test accuracy on n = 919, the 95% confidence interval for accuracy is [0.934, 0.962], computed with the Wilson score interval for a single proportion^53,54^. These intervals quantify sampling uncertainty on the fixed held-out test set. They do not quantify variability due to stochastic neural-network training choices such as random initialization, mini-batch ordering, or optimizer randomness. Accordingly, the present results should be interpreted as single-split performance estimates under the stated evaluation protocol, and multi-seed repetition remains an important next step for assessing training stability. Moreover, the slope trend depicted in Fig. 6 emphasizes the increasing success rate as more training epochs were completed, with performance stabilizing beyond epoch 30.

The model’s strength lies in its accuracy and its ability to handle high-dimensional, correlated financial data, a common challenge in enterprise analytics. The combination of automated feature extraction, robust classification, and statistical consistency makes this model a useful addition to enterprise business intelligence systems^8,37,39^.

## Conclusion

This study presents a deep learning-based approach to improve the accuracy and reliability of enterprise financial data by using CNNs. The model combines AI with enterprise information systems and big data and helps businesses detect anomalies and prioritize records for auditable finance review more efficiently, supporting controlled correction workflows within EMIS.

Experiments, based on a regional dataset spanning two decades, showed that the CNN model performed strongly under the present evaluation design. It outperformed the classical baseline models included in this study, namely SVM, NB, and LDA, and delivered strong metrics in terms of accuracy, G-mean, MCC, and AUC. One key advantage of using CNNs is their ability to automatically learn features from data, reducing the need for manual preprocessing and making the system more scalable.

Dataset balance was improved through SMOTE, which improved the model’s overall generalization and performance. These findings suggest that AI-powered tools can make a real difference in how businesses manage financial information, detect anomalies, and plan ahead.

That said, there are still some limitations in this study. For instance, the current model is mainly designed for structured, historical data. It may need further refinement to work effectively with real-time or unstructured inputs. Another challenge is interpretability, since deep learning models are often perceived as “black boxes,” which can reduce confidence for financial controllers, auditors, and compliance stakeholders. In addition, because the CNN operates on a predefined finance-informed indicator sequence, robustness to alternative feature orderings was not evaluated in the present study and remains an important direction for future validation. An additional empirical limitation is that the comparative evaluation did not yet include strong modern tabular ensemble baselines such as XGBoost, LightGBM, and CatBoost; therefore, the present study should not be interpreted as establishing CNN superiority over the current strongest family of tabular learners. Finally, the reported confidence intervals quantify sampling uncertainty on the fixed held-out test set and do not substitute for multi-seed retraining when assessing neural-model stability.

Enterprise deployment also requires practical operational steps beyond model accuracy. A large organization would typically implement (i) automated ETL/ELT pipelines from ERP/CRM/data warehouse sources into a governed feature store, (ii) model serving as a secured application-layer service within the cloud architecture (with versioning, access control, and latency monitoring), (iii) continuous monitoring for data drift and performance decay across reporting periods, and (iv) immutable audit logging so that each flagged record is traceable to the exact model version and input snapshot that produced the decision. This operational framing supports controlled “correction workflows” where finance staff validate anomalies and approve record status changes within EMIS, rather than unreviewed automatic modification of accounting data.

Future work can focus on integrating explainable AI (XAI) to help users understand and audit model decisions made by the system. For tabular finance indicators and enterprise governance, local and global explanation methods such as SHAP and LIME can provide feature-attribution summaries that are suitable for audit trails and stakeholder review^55,56^. For the neural model specifically, gradient-based attribution methods such as Integrated Gradients and DeepLIFT can be used to generate consistent indicator-level contribution scores for individual decisions^57,58^. These explanation artifacts can be stored alongside model outputs inside EMIS to support auditability and compliance reporting. Other possible directions include adapting the model for multi-class error detection, integrating it more tightly with ERP or CRM correction workflows, and validating it across different industries and regulatory environments.

In summary, this research shows how AI, particularly CNNs, can bring greater intelligence, precision, and automation to enterprise financial management. It offers a solid step toward smarter, more responsive systems in the age of data-driven business.

This study was limited to structured, historical financial data, which may not fully represent real-time or unstructured financial transactions. Moreover, the model’s predictive accuracy depends on the quality of enterprise datasets, which may vary across industries. Future research should extend this framework by incorporating explainable-AI techniques and real-time big data streams to improve interpretability and adaptability.

## Data Availability

The datasets generated and/or analysed during the current study are not publicly available due to institutional data-use restrictions but are available from the corresponding author on reasonable request.
